# Integrated spectral and depth compensation approach for optimizing oxygen saturation and total hemoglobin estimation in photoacoustic tomography for ovarian lesion diagnosis

**DOI:** 10.1117/1.JBO.31.2.026002

**Published:** 2026-02-04

**Authors:** Lukai Wang, Yixiao Lin, Haolin Nie, Jinhua Xu, Sanskar Thakur, Quing Zhu

**Affiliations:** a Washington University in St. Louis, Imaging Science Program, St. Louis, Missouri, United States; b Washington University in St. Louis, Department of Biomedical Engineering, St. Louis, Missouri, United States; c Washington University School of Medicine, Department of Radiology, St. Louis, Missouri, United States

**Keywords:** photoacoustic tomography, oxygen saturation, total hemoglobin concentration, fluence compensation, spectral coloring effect, ovarian cancer diagnosis, functional imaging

## Abstract

**Significance:**

Photoacoustic tomography (PAT) holds promise for non-invasive functional imaging in ovarian cancer diagnostics. However, accurate estimation of oxygen saturation (${\%\mathrm{sO}}_{2}$) and total hemoglobin concentration (THb) is hindered by wavelength- and depth-dependent fluence variations.

**Aim:**

We aim to improve the accuracy and clinical utility of ${\%\mathrm{sO}}_{2}$ and THb quantification in transvaginal ultrasound-guided PAT (US-PAT) by developing an integrated spectral and depth compensation (ISDC) method that corrects for both spectral distortion and depth-dependent attenuation.

**Approach:**

We introduce a spectral compensation strategy derived from Monte Carlo simulations and integrate it with depth-wise fluence correction to construct the proposed ISDC method. The approach has been validated using phantoms with known optical properties and applied to clinical PAT data from 82 ovarian lesions (67 benign and 15 malignant). Diagnostic performance was evaluated using logistic regression and receiver operating characteristic analysis.

**Results:**

In phantom experiments, ISDC improved ${\%\mathrm{sO}}_{2}$ estimation accuracy compared with linear unmixing (LU) and enhanced uniformity of THb estimates across depth. In clinical data, ISDC has increased ${\%\mathrm{sO}}_{2}$ values by ∼5% in both benign and malignant lesions, enhanced contrast of THb between malignant and benign lesion groups (mean THb ratio $R_{\mathrm{THb}}$ has increased from 1.4 to 1.9), and achieved higher classification performance (AUC = 0.93 versus 0.88 for LU) when combining ${\%\mathrm{sO}}_{2}$ and THb features.

**Conclusions:**

The ISDC approach significantly enhances the quantitative accuracy and diagnostic performance of PAT by compensating for both spectral and depth fluence variations within biological tissue. These improvements support the integration of ISDC into US-PAT systems for ovarian lesion characterization and future clinical applications.

## 1 Introduction

Photoacoustic tomography (PAT) combines optical absorption contrast with ultrasound resolution to provide high-resolution and deep-tissue imaging of biological structures and functions.^1^ Multispectral PAT further extends this capability by enabling the calculation of quantitative metrics, such as oxygen saturation (${\%\mathrm{sO}}_{2}$) and total hemoglobin concentration (THb).^2^ Conventionally, ${\%\mathrm{sO}}_{2}$ and THb estimation relies on linear unmixing (LU), which assumes a wavelength-independent fluence profile and a depth-invariant fluence distribution.^3^ However, wavelength-dependent light–tissue interactions, such as light absorption and scattering, introduce nonlinearities to the fluence distribution, leading to both wavelength-dependent and depth-dependent variations that affect accurate quantification of ${\%\mathrm{sO}}_{2}$ and THb. The wavelength-dependent fluence variation results in the spectral coloring effect, where the assumption of a linear relationship between optical absorption and the corresponding photoacoustic amplitude is violated, causing systematic errors in ${\%\mathrm{sO}}_{2}$ estimation with the conventional LU method. Meanwhile, depth-dependent fluence attenuation reduces the overall signal intensity with depth, further limiting the accuracy of quantitative PAT, especially in the estimation of THb. Over the years, various methods have been proposed to address these challenges. Some methods that address the spectral coloring effect are eigenspectral analysis,^4^ machine learning^5^ and deep learning neural network,^6^ Bayesian approach,^7^ intervascular ${\%\mathrm{sO}}_{2}$ difference estimation,^8^ and analytical Monte Carlo method.^9^^,^^10^ Depth-dependent fluence attenuation has been addressed using the Beer–Lambert law,^11^ Monte Carlo-based correction in heterogeneous media,^12^ and iterative inverse problem formulations that model depth-wise attenuation for accurate fluence compensation.^13^ However, most existing methods are not easily applicable in clinical settings because they generally have low generalizability and high computational costs. Therefore, a practical and computationally efficient approach that corrects both spectral and depth-dependent fluence variations is needed to improve the robustness of quantitative PAT in clinical applications.

Our group has pioneered the use of ${\%\mathrm{sO}}_{2}$ and THb derived from PAT for ovarian cancer diagnosis.^2^^,^^14^^–^^16^ Our objective in this study is to develop a simple yet effective approach to improve ${\%\mathrm{sO}}_{2}$ and THb estimations in our transvaginal co-registered ultrasound (US) and PAT (US-PAT) system by addressing both spectral and depth-dependent fluence variations. For spectral compensation, we implemented two physics-based methods derived from Monte Carlo simulations: the convex cone (CC) and compensated linear unmixing (CLU) methods. The CC method incorporates fluence eigenspectra obtained from Monte Carlo simulations,^10^ whereas the CLU method applies a compensatory fluence spectrum to correct for wavelength-dependent fluence variations in LU. To further enhance THb estimation, we integrated depth-wise fluence compensation. By leveraging a Monte Carlo simulation of a simplified digital phantom, we propose an integrated spectral and depth compensation (ISDC) strategy that combines both CLU and depth-wise fluence compensation to enhance both ${\%\mathrm{sO}}_{2}$ and THb estimation while maintaining computational efficiency.

In this study, we first validated the ISDC approach in phantom experiments by comparing CC and CLU with the conventional LU method. Both spectral and depth-wise fluence compensation were incorporated to improve accuracy. Given the improved accuracy and efficiency of CLU with fewer wavelengths, we selected CLU for ISDC implementation in ovarian patient data. Applying ISDC in patient data improved differentiation between malignant and benign lesions and yielded ${\%\mathrm{sO}}_{2}$ estimates that better aligned with physiological expectations.

## 2 Materials and Methods

### 2.1 Co-registered US-PAT Imaging System

**Fig. 1:**
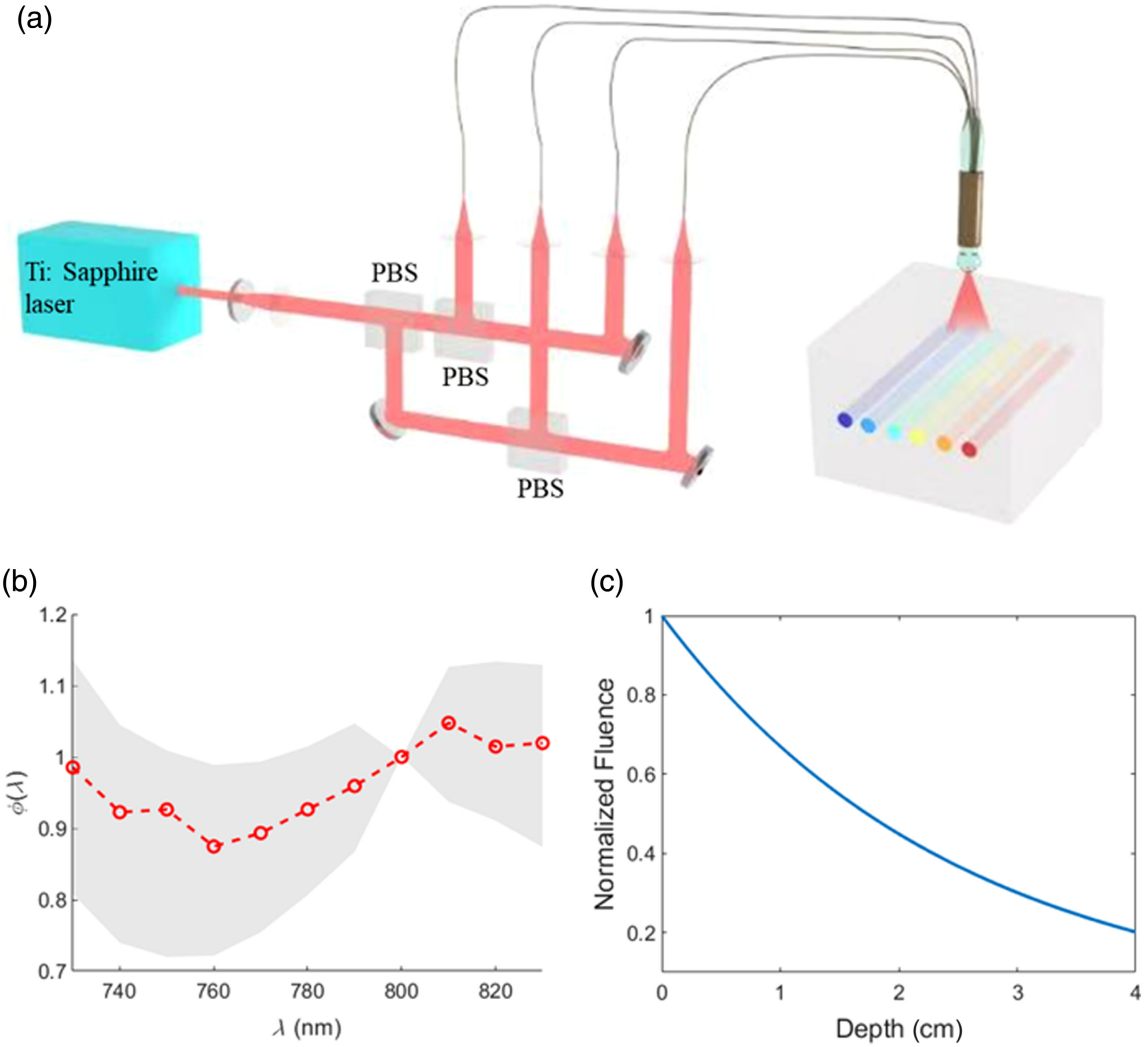
(a) Schematic of the US-PAT illumination system used to deliver light into a digital phantom. Pulsed light from a tunable Ti:sapphire laser is evenly split into four optical fibers via a compact optical system employing three polarizing beam splitters. Two fibers on each side of the ultrasound (US) probe provide illumination. The US probe and fibers are enclosed within a thin resin sheath coated internally with a diffuse reflective layer to enhance illumination uniformity. In the digital phantom, the gray region represents the background medium, and the red tubular structures denote absorbing chromophores. (b) Normalized fluence spectra ϕ(λ) generated from Monte Carlo simulations for the CC method. The shaded region represents the set of normalized fluence spectra used to construct the convex cone, and the red dashed line denotes their average spectrum $\phi_{\mathrm{ave}}(\lambda )$, which is incorporated into the CLU method for spectral compensation. (c) Depth-wise fluence attenuation profile (normalized) demonstrating exponential decay with increasing tissue depth, used for depth compensation in the ISDC approach.

Imaging was conducted using the previously described transvaginal US-PAT system.^14^^,^^15^ In this setup, PAT signals are excited by a tunable Ti:sapphire pulsed laser (LOTIS TII, Minsk, Belarus) delivering light to the tissue via four optical fibers, as illustrated in Fig. 1(a). Co-registered US-PAT scans were performed using a commercial transvaginal ultrasound system (E-CUBE 12, Alpinion Medical Systems, Seoul, Republic of Korea). PAT data can be acquired at custom wavelengths within the range of 730 to 870 nm, encompassing the isosbestic point of oxy- and deoxyhemoglobin at 808 nm, using our custom-developed US-PAT display software.^17^ For the clinical ovarian lesion dataset, images were acquired at 750, 780, 800, and 830 nm. To enhance the signal-to-noise ratio, images at each wavelength were averaged five times. Data acquisition at these wavelengths was performed sequentially and interleaved with real-time ultrasound B-mode imaging, with ∼4  s required to acquire data at each wavelength. In addition, the interleaved beamforming of US and PAT enabled real-time, co-registered US-PAT visualization during imaging sessions.

### 2.2 ${\%\mathrm{sO}}_{2}$ and THb Calculation Methods

Conventionally, the LU method is used to estimate ${\%\mathrm{sO}}_{2}$ and THb. The key to this process is extracting the local absorption spectrum $\mu_{a}(R,\lambda )$ from the measured local acoustic pressure spectrum $p_{0}(R,\lambda )$ and determining the local concentrations of oxy- and deoxy-hemoglobin $c_{\mathrm{ox}}(R)$ and $c_{\mathrm{de}}(R)$ by linear fitting

$$\mu_{a}(R,\lambda )=c_{\mathrm{de}}(R)\varepsilon_{\mathrm{de}}(\lambda )+c_{\mathrm{ox}}(R)\varepsilon_{\mathrm{ox}}(\lambda ),$$ (1)

where $\varepsilon_{\mathrm{ox}}(\lambda )$ and $\varepsilon_{\mathrm{de}}(\lambda )$ are the molar extinction coefficients spectra of oxy- and deoxy-hemoglobin, respectively. R(x,y,z) is the target location in 3D or R(x,z) in 2D. To ensure physiologically meaningful results, a non-negativity constraint is applied to $c_{\mathrm{ox}}(R)$ and $c_{\mathrm{de}}(R)$ during the linear fitting process. $p_{0}(R,\lambda )$ is calculated from radio-frequency data with the delay-and-sum method, and its relationship with $\mu_{a}(R,\lambda )$ is given by

$$p_{0}(R,\lambda )=\Gamma (R)\phi (R,\lambda )\mu_{a}(R,\lambda ),$$ (2)

where ϕ(R,λ) represents the local fluence spectrum, and Γ(R) is the Grüneisen coefficient, which is independent of λ. The conventional LU method assumes a wavelength-independent fluence profile^3^ so that $\mu_{a}(R,\lambda )$ can be directly recovered from $p_{0}(R,\lambda )$ using the proportionality: $p_{0}(R,\lambda )\propto \mu_{a}(R,\lambda )$. The corresponding local ${\%\mathrm{sO}}_{2}$ and THb are then computed as

$$\%{\mathrm{sO}}_{2}(R)=\frac{c_{\mathrm{ox}}(R)}{c_{\mathrm{ox}}(R)+c_{\mathrm{de}}(R)}\times 100\%,$$ (3)

$$\mathrm{THb}(R)=c_{\mathrm{ox}}(R)+c_{\mathrm{de}}(R).$$ (4)

The CC method is a physics-driven approach that models wavelength-dependent fluence variations using eigenspectra derived from Monte Carlo simulations of digital phantoms.^9^ In this framework, wavelength-dependent Monte Carlo simulations^18^ were performed on simplified digital phantoms containing key absorbers, such as hemoglobin, copper sulfate, and nickel sulfate, embedded in a surrounding scattering medium. Simulations were conducted across multiple wavelengths, incorporating the respective optical absorption and scattering coefficients, with the light source configured to match the illumination geometry of our US-PAT system. The resulting wavelength-dependent fluence distributions were subjected to dimension reduction to obtain a set of fluence eigenspectra $\left\{\phi_{1}(\lambda ),\phi_{2}(\lambda ),\ldots ,\phi_{n}(\lambda )\right\}$, where n is the number of wavelength that captures the dominant modes of spectral variation, as shown in Fig. 1(b). The measured photoacoustic spectrum $p_{0}(\lambda )$ depends on both the absorption spectrum and the local fluence spectrum ϕ(R,λ). The latter is represented as a non-negative linear combination of the eigenspectra

$$\phi (R,\lambda )=\overset{n}{\underset{i=1}{\sum }}\alpha_{i}\phi_{i}(\lambda ),\;\alpha_{i}\geq 0.$$ (5)

The eigenspectra form a convex cone in a high-dimensional spectral space. ${\%\mathrm{sO}}_{2}$ is estimated by minimizing the angular distance between the measured photoacoustic spectrum $p_{0}(R,\lambda )$ and the cones—achieving high accuracy even under spectral distortions. This approach is robust because fluence spectra from simple digital phantoms can effectively capture photon absorption and scattering behavior in more complex experimental conditions. Although prior knowledge of tissue optical properties is required, the CC method is tolerant to moderate misestimation, making it broadly applicable to heterogeneous and poorly characterized media.

**Fig. 2:**
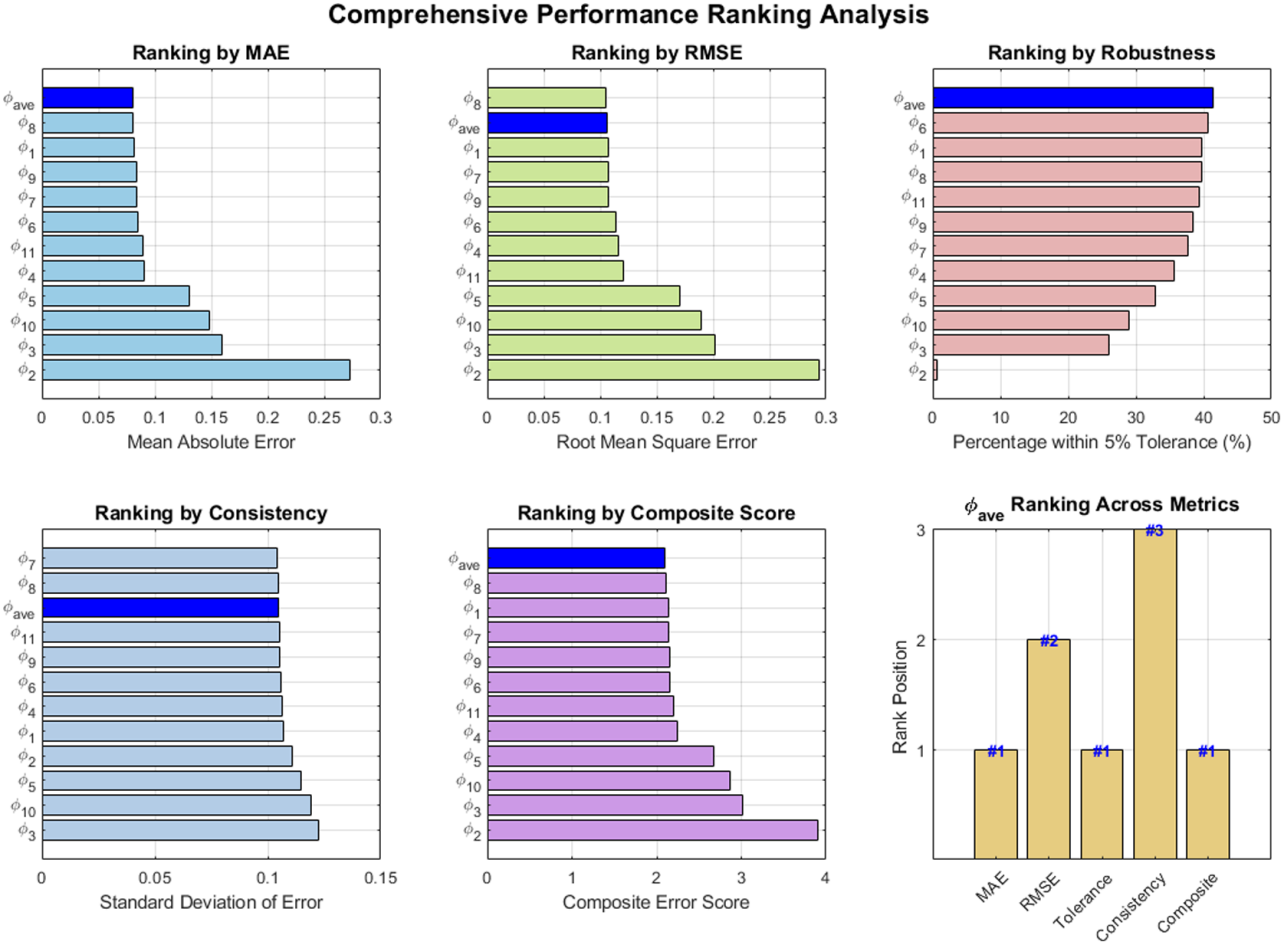
Comprehensive numerical validation of the average fluence spectrum ($\phi_{\mathrm{ave}}$) as the optimal single-spectrum compensator. Performance ranking of CLU compensated by individual fluence eigenspectra ($\phi_{1}$ to $\phi_{11}$) versus the proposed $\phi_{\mathrm{ave}}$ across multiple metrics using a digital phantom: (a) MAE. (b) RMSE. (c) Robustness (percentage within 5% tolerance). (d) Consistency (standard deviation of error). (e) Composite error score summarizing all metrics. (f) Overall ranking of $\phi_{\mathrm{ave}}$ across all metrics. Across MAE, robustness, and composite score, $\phi_{\mathrm{ave}}$ consistently attains rank 1, confirming it as the statistically optimal and most reliable single-spectrum compensation strategy compared with using any individual eigenspectrum.

Although CC provides a rigorous and adaptive representation of wavelength-dependent fluence, its accuracy relies on having a sufficient number of sampled wavelengths, which can be challenging in clinical imaging due to constraints such as imaging speed, motion artifacts, and patient comfort. To address these limitations while retaining the core advantages of CC, we developed the CLU method as a simplified and computationally efficient approximation of CC. CLU incorporates a single, representative compensating spectrum, defined as the ensemble mean of the normalized eigenspectra, into the LU process

$$p_{0}(R,\lambda )\propto \mu_{a}(R,\lambda )\phi_{\mathrm{ave}}(\lambda ),$$ (6)

where $\phi_{\mathrm{ave}}(800\;\mathrm{nm})$ is normalized to 1 for practical implementation because 800 nm is close to the isosbestic point of hemoglobin. The rationale for using the ensemble mean $\phi_{\mathrm{ave}}(\lambda )$ is mathematically derived in detail in the Supplementary Material. To further evaluate the effectiveness of $\phi_{\mathrm{ave}}(\lambda )$, we conducted a numerical experiment using the Monte Carlo simulated fluence data of the digital phantom corresponding to Fig. 1(b). Specifically, we compared CLU performance using $\phi_{\mathrm{ave}}(\lambda )$ against CLU performance using each of the 11 individual fluence eigenspectra ($\phi_{1}$ to $\phi_{11}$) derived from the Monte Carlo set across the 730- to 830-nm wavelength range (11 wavelengths total). The key results are shown in Fig. 2 and described as follows:

• Accuracy [mean absolute error (MAE) and root mean square error (RMSE)]: CLU using $\phi_{\mathrm{ave}}$ achieved rank 1 in MAE and rank 2 in RMSE, indicating that it provides the lowest mean error on average while maintaining near-best performance in L2 error compared with all individual eigenspectra.
• Robustness and consistency: $\phi_{\mathrm{ave}}$ achieved rank 1 in robustness and rank 2 in consistency, demonstrating both strong reliability across diverse conditions and low error variability comparable to the best-performing individual spectrum.
• Overall performance (composite score): When considering a composite score that combines MAE, RMSE, consistency, and robustness, $\phi_{\mathrm{ave}}$ again secured rank 1. This confirms it as the best overall compromise across all performance metrics.

Collectively, these results show that $\phi_{\mathrm{ave}}$ is a statistically optimal and reliable single-spectrum compensator. Although some individual eigenspectrum may perform well in isolated metrics, their performance is highly variable and context-dependent. In contrast, $\phi_{\mathrm{ave}}$ consistently provides top-tier accuracy, stability, and robustness, making it the superior choice for CLU under limited spectral sampling and realistic noise conditions.

As CLU directly modifies the fluence spectrum in the linear unmixing framework, it requires fewer wavelengths than CC to achieve stable ${\%\mathrm{sO}}_{2}$ estimations. Given these characteristics, we used both CC and CLU in our phantom experiments, considering CC as a reference standard due to its higher theoretical accuracy when multiple wavelengths are available. Although CLU within ISDC can theoretically operate with as few as two wavelengths, at least three to four wavelengths are required to ensure a robust unmixing process against system noise and measurement errors and to reliably estimate ${\%\mathrm{sO}}_{2}$.

In addition to spectral compensation, depth-wise compensation was applied to correct for signal attenuation with increasing depth. This attenuation arises from optical fluence decay due to photon propagation to the target and acoustic attenuation of the generated photoacoustic wave when it propagates from the target to the transducer. We accounted for both effects by modeling the fluence decay and acoustic attenuation using exponential functions. The fluence attenuation coefficient, $\mu_{f}$, was estimated from simulation data as shown in Fig. 1(c), whereas the acoustic attenuation coefficient, $\mu_{b}$, was derived from corresponding co-registered US measurements. These coefficients were combined to perform depth-wise compensation as

$$p_{0,c}(R,\lambda )=\overset{˜}{p_{0}}(R,\lambda )\exp[(\mu_{f}+\mu_{b})R],$$ (7)

where $p_{0,c}(R,\lambda )$ and $\overset{˜}{p_{0}}(R,\lambda )$ are the compensated and beamformed local pressure at R, respectively. Scalar R is the distance from the field point to the transducer and approximated by depth z because x is much smaller than the depth dimension in phantom and clinical imaging. The compensated pressure $p_{0,c}(R,\lambda )$ is then applied for THb calculation in conjunction with spectral compensation.

### 2.3 Phantoms

To evaluate the performance of the ISDC approach, we prepared four depth-varying tube phantoms containing nickel sulfate (${\mathrm{NiSO}}_{4}$) and copper sulfate (${\mathrm{CuSO}}_{4}$) solutions. ${\mathrm{NiSO}}_{4}$ and ${\mathrm{CuSO}}_{4}$ are widely used in photoacoustic imaging phantoms due to their similarity in optical absorption spectra to oxy- and deoxy-hemoglobin as well as their chemical stability, making them effective surrogates for blood optical properties.^19^ The nickel saturation %sNi, an analog to ${\%\mathrm{sO}}_{2}$, is defined as

$$\%\mathrm{sNi}=\frac{\frac{C_{{\mathrm{NiSO}}_{4}}}{14.28}}{\frac{C_{{\mathrm{NiSO}}_{4}}}{14.28}+C_{{\mathrm{CuSO}}_{4}}}\times 100\%,$$ (8)

where $C_{{\mathrm{NiSO}}_{4}}$ and $C_{{\mathrm{CuSO}}_{4}}$ represent the concentrations of ${\mathrm{NiSO}}_{4}$ and ${\mathrm{CuSO}}_{4}$, respectively. The factor of 14.28 accounts for the higher absorption coefficient of ${\mathrm{CuSO}}_{4}$ compared with ${\mathrm{NiSO}}_{4}$, ensuring an appropriate scaling for %sNi estimation. Similarly, we define total sulfate concentration TSf as an analog to THb as

$$\mathrm{TSf}=\frac{C_{{\mathrm{NiSO}}_{4}}}{14.28}+C_{{\mathrm{CuSO}}_{4}}.$$ (9)

**Fig. 3:**
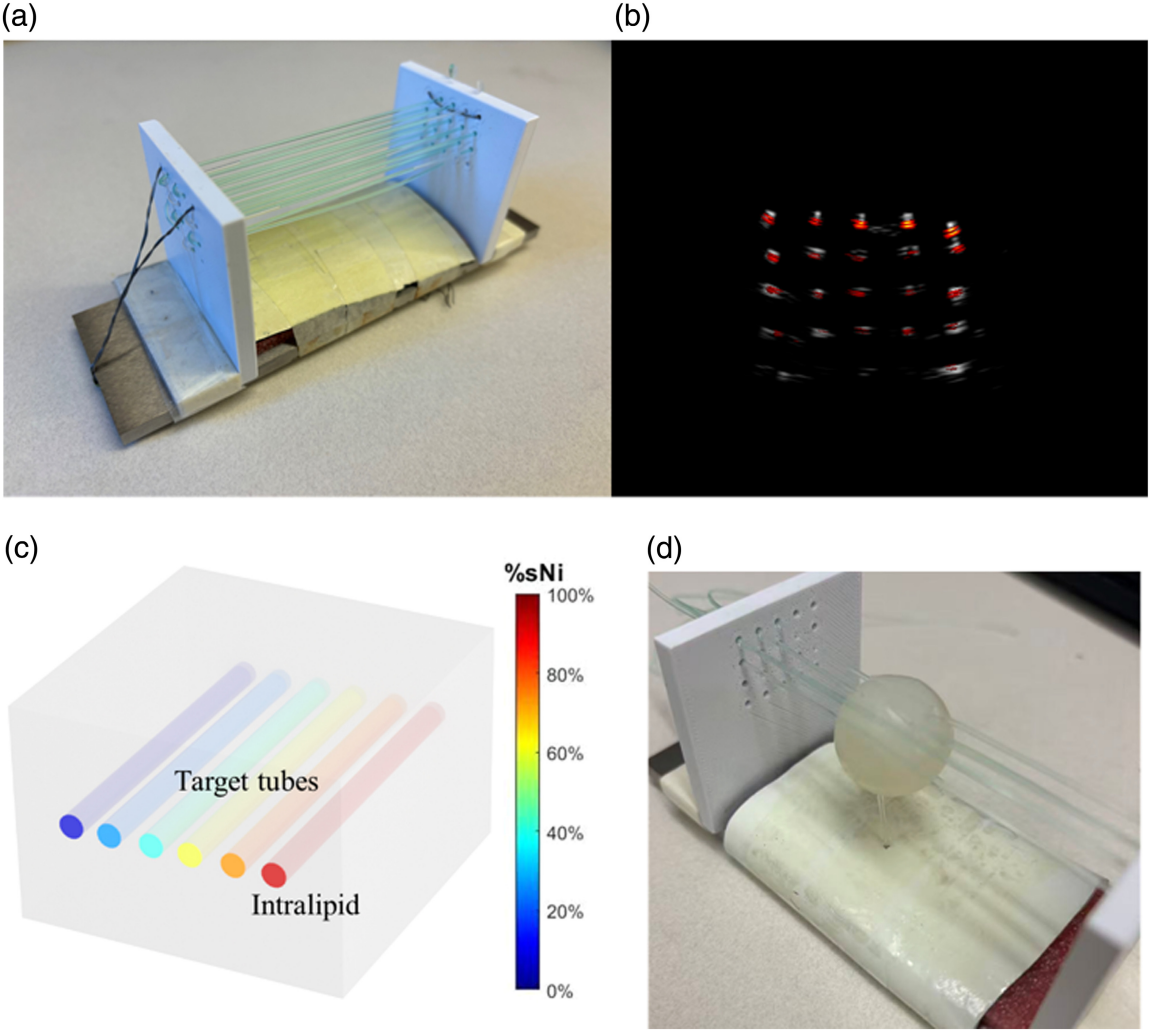
(a) Depth-varying tube phantom with embedded tubes positioned at varying depths. (b) Co-registered US-PAT image of the depth-varying tube phantom. (c) Digital phantom for simulations, illustrating target tubes embedded in an Intralipid medium with various %sNi values. (d) Agar-based spherical phantom mimicking ovarian tissue.

For this study, we prepared phantoms with %sNi values of 20%, 60%, 80% and 100%, enabling a comprehensive evaluation of the method. Each phantom contained a tube filled with the sulfate solution, which was threaded into a 2D rectangular point grid and secured using two panels with pre-drilled holes, as illustrated in Fig. 3(a). This setup allowed one tube to be positioned at varying depths and elevations within a single phantom, ensuring that TSf remained uniform within each phantom.

After preparation, the phantoms were immersed in an Intralipid solution with a calibrated reduced scattering coefficient of $∼1{\;\mathrm{cm}}^{-1}$. The US-PAT system acquired data at wavelengths from 730 to 830 nm in 10-nm increments. Monte Carlo simulations of a corresponding digital phantom were used to generate the fluence eigenspectra and model depth-dependent fluence attenuation.

ISDC was then validated on an ovary-mimicking phantom to emulate the complex imaging conditions in a clinical setting. The phantom consisted of an agar sphere (∼2  cm diameter) to simulate the ovary. It was mixed with glass microspheres for controlled calibration of its optical and acoustic scattering. The phantom consisted of six blood vessels simulated by tubes: four tubes (two at %sNi=40% and two at %sNi=75%) passed through the agar sphere to mimic vessels inside the ovary, whereas two additional tubes (one at %sNi=40% and one at %sNi=75%) were positioned outside the sphere to represent adjacent vasculature.

During experiments, the entire phantom was immersed in an Intralipid solution with a calibrated reduced scattering coefficient of $∼1\;{\mathrm{cm}}^{-1}$, ensuring controlled, homogeneous background scattering. This configuration provided a combination of large-volume spectral coloring, irregular target geometry, and spatially varying fluence, enabling a more realistic assessment of ISDC performance under conditions that better approximate the optical complexity of ovarian tissue.

### 2.4 Patient Data

The study protocol was approved by the Institutional Review Board. All patients signed the informed consent. A total of 60 patients were included in this study. Among them, 15 malignant and 67 benign ovarian masses were identified, with some patients having bilateral lesions of the same or differing pathologies. The US-PAT system was used to image patients’ ovaries at wavelengths of 750, 780, 800, and 830 nm. A reduced number of wavelengths was selected to minimize the impact of motion during patient imaging and to expedite the acquisition process.

## 3 Results

### 3.1 Phantom Results

**Fig. 4:**
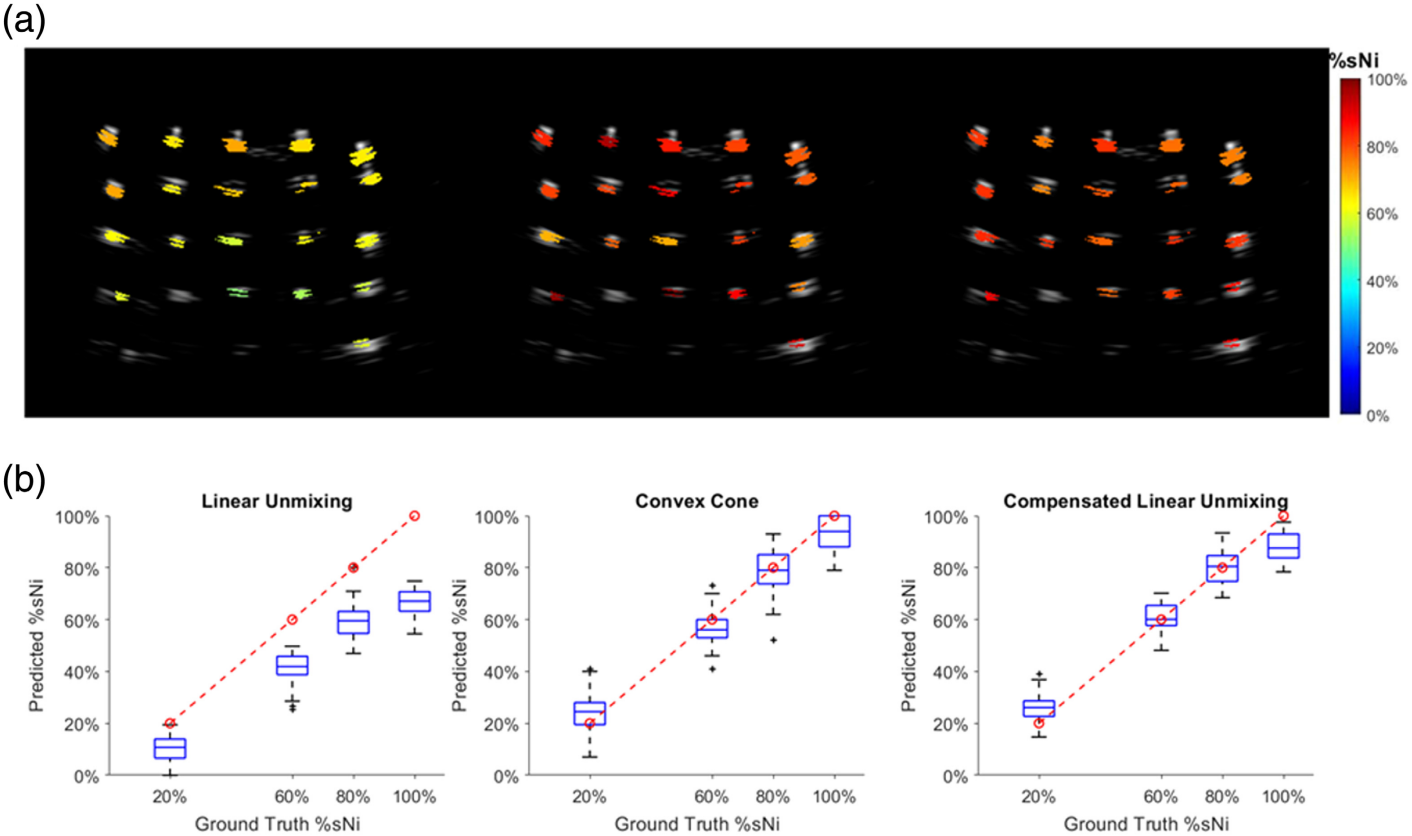
(a) Representative co-registered US-%sNi images of a depth-varying tube phantom with a ground truth %sNi of 80%, computed using LU, CC, and CLU methods. (b) Comparison of %sNi calculations for phantom data using LU, CC, and CLU methods. The red dashed line represents the ground truth.

The results of %sNi estimation for the depth-varying tube phantoms are presented in Fig. 4. Due to system noise and measurement errors, as well as the limitation that the pixel size is smaller than the theoretical resolution, calculating %sNi at each individual pixel may not yield robust results. To mitigate this problem, we implemented a clustering method, where pixels within each tube cross-section are grouped into clusters, and the average $p_{0}$ spectrum for the cluster was calculated and used for %sNi estimation. Each B-scan contained ∼15 cross-sections. For each %sNi value, data from 4 B-scans are used, resulting in ∼60 tube cross-sections per %sNi value.

As shown in Fig. 4(b), LU consistently underestimates %sNi compared with the ground truth with a relatively constant error across different ground truth values. This supports the idea that the spectral coloring effect introduces a systematic bias.^8^ In contrast, both CC and CLU provide more accurate estimations by accounting for the nonlinear effects of optical scattering and absorption, resulting in %sNi values closer to the ground truth than LU.

Between the two methods, CLU demonstrated better stability than CC, as evidenced by its smaller interquartile ranges and fewer outliers. This improved stability is likely due to its higher tolerance to system noise and measurement errors with a limited number of wavelengths, which reduces the sensitivity of the unmixing process to the reconstruction variability in the $p_{0}$ spectra. Although CC showed slightly better accuracy for the %sNi=100% phantom, the overall performance of CC and CLU remains comparable, reinforcing the feasibility of CLU as a more practical alternative in cases where wavelength constraints and computational efficiency are considerations.

**Fig. 5:**
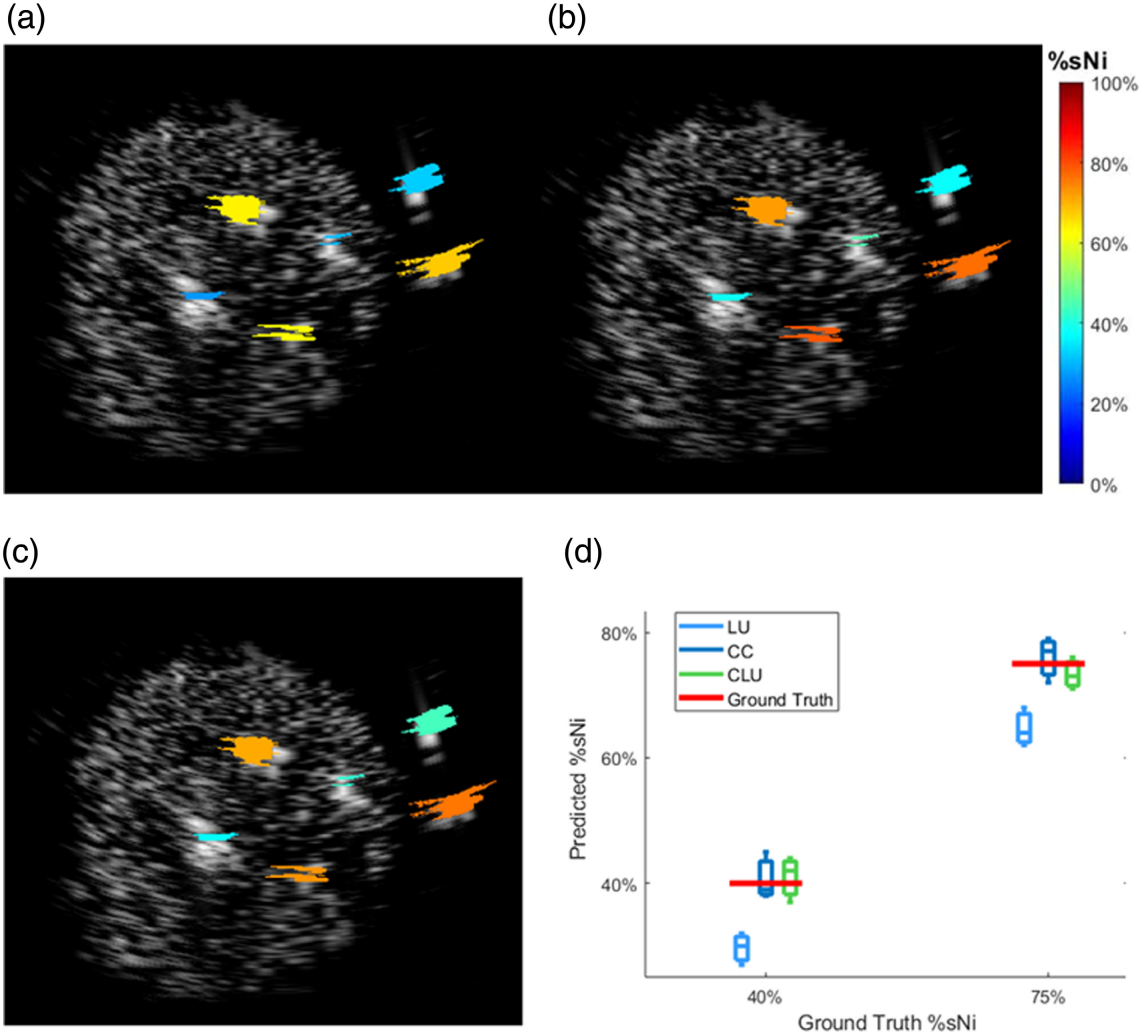
Co-registered US-%sNi images from the spherical phantom using (a) LU, (b) CC, and (c) CLU. (d) Predicted %sNi values for 40%- and 75%–%sNi tubes, with dots representing individual tubes and red lines indicating ground truth.

The results of %sNi estimation for the agar-based spherical phantom are shown in Fig. 5. LU underestimated %sNi at both ground truth levels, whereas both CC and CLU substantially improved accuracy and lowered spatially dependent variation even in the presence of a more complex scattering environment. The quantitative comparison in Fig. 5(d) shows that CLU closely matches the CC reference and maintains accuracy for both %sNi levels, confirming that CLU remains effective even under more complex, tissue-like conditions.

These results validate that CC and CLU can be generalized beyond simple tube phantoms in Intralipid solutions and remain robust in the presence of complex scattering structures. The consistency across tubes placed at different depths and positions indicates that the proposed method can robustly correct spectral coloring arising from volumetric tissue heterogeneity, thereby supporting its applicability to in-vivo ovarian imaging.

**Fig. 6:**
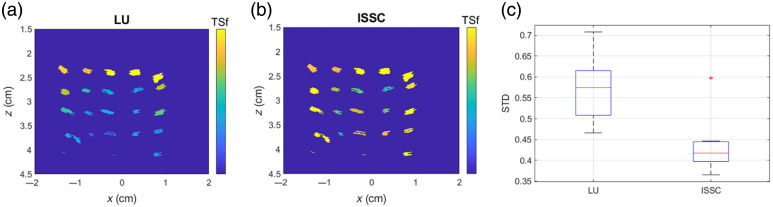
Example of TSf estimation using (a) LU and (b) ISDC approach in a tube phantom. (c) Distribution of TSf standard deviation across 16 B-scans from four phantoms, where the STD is calculated from the TSf values of cross-sections within each B-scan.

Furthermore, we applied the ISDC approach with CLU-based spectral compensation to TSf estimation and compared it with the conventional LU-based method. Ideally, TSf should be uniform across all observed tube cross-sections within a B-scan, as they originate from a single continuous tube that is intertwined around the two panels. As shown in Fig. 6(a) and Fig. 6(b), LU leads to a depth-dependent decline in TSf values, whereas the ISDC approach enhances uniformity across different depths. To quantify TSf uniformity within each B-scan, we calculated the standard deviation (STD)

$$\mathrm{STD}=\sqrt{\frac{1}{n}\overset{n}{\underset{i=1}{\sum }}{\left({\mathrm{TSf}}_{i}-\overset{¯}{\mathrm{TSf}}\right)}^{2}},$$ (10)

where n is the number of cross-sections within the B-scan, ${\mathrm{TSf}}_{i}$ represents the TSf of each cross-section, and $\overset{¯}{\mathrm{TSf}}$ represents the mean TSf value. Figure 6(c) summarizes the STD of TSf estimation across 16 B-scans from 4 tube phantoms. The results indicate that the ISDC approach significantly improves the uniformity of TSf estimation.

### 3.2 Patient Results

As shown in the phantom experiments, CLU offers greater stability with fewer wavelengths and is computationally efficient, making it a practical choice for real-time analysis. Therefore, we implemented the ISDC approach with CLU as the correction method for our ovarian patient data. The ${\%\mathrm{sO}}_{2}$ and THb levels in ovarian tissues have been previously reported in our publications,^2^^,^^9^ showing that benign lesions have higher ${\%\mathrm{sO}}_{2}$ values and lower THb values compared with malignant lesions.

**Fig. 7:**
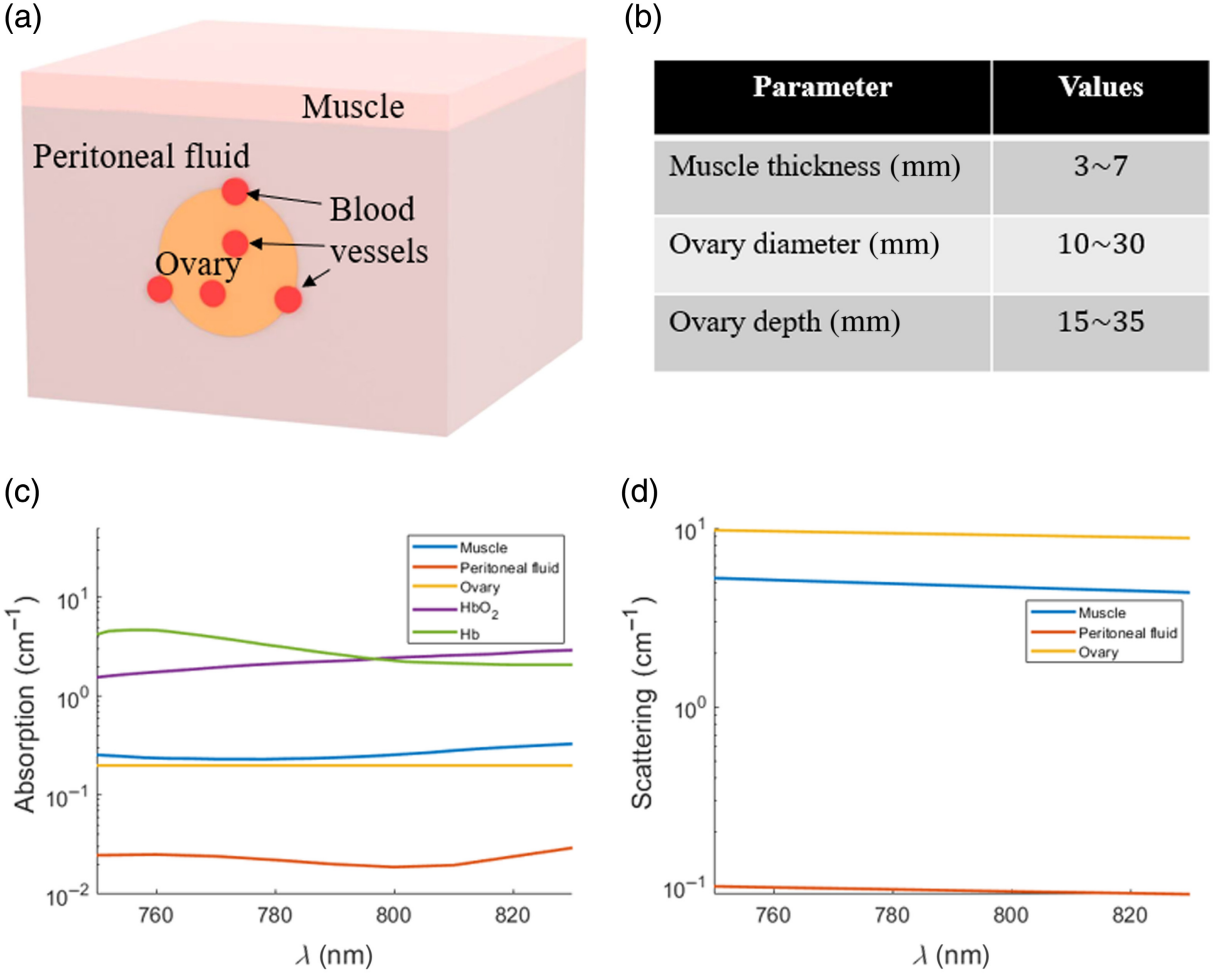
(a) Schematic of a representative digital phantom comprising muscle, peritoneal fluid, ovary, and embedded blood vessels. (b) Parameter ranges used in constructing the digital phantom. (c) Absorption coefficients and (d) reduced scattering coefficients of the individual tissue components used in the digital phantom. ${\mathrm{HbO}}_{2}$ and Hb are modeled as pure absorbers, and their scattering contributions are not considered in the simulation.

The primary challenge in studying ovarian ${\%\mathrm{sO}}_{2}$ and THb is the absence of a definitive ground truth. As these parameters are valuable for distinguishing malignant from benign lesions, we evaluated their performance based on their ability to differentiate between the two groups, offering a practical framework for assessing diagnostic utility. To enable fluence compensation with the ISDC approach, several digital phantoms were constructed to mimic the anatomical and optical environment encountered during transvaginal US-PAT imaging. Each phantom incorporated key tissue layers, including a superficial muscle layer, peritoneal fluid, and an embedded ovary with blood vessels. Figure 7(a) presents a representative digital phantom, and the geometrical metrics are summarized in Fig. 7(b). The compensating fluence spectra were generated using Monte Carlo simulations informed by documented optical properties of human tissues,^20^^–^^23^ as shown in Figs. 7(c) and 7(d).

**Fig. 8:**
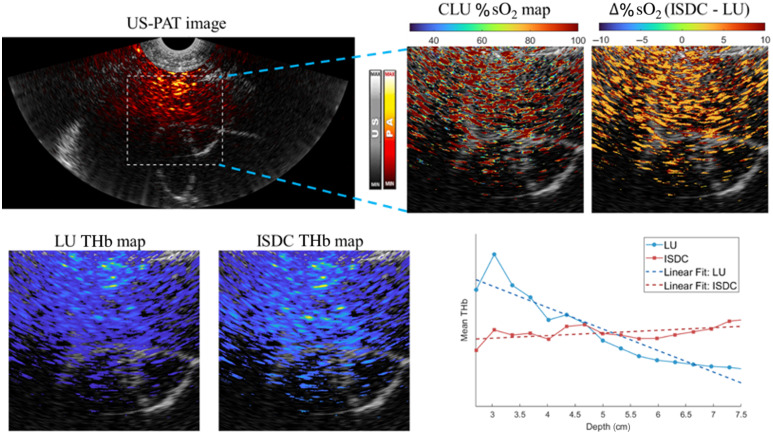
Comparison of ${\%\mathrm{sO}}_{2}$ and THb maps obtained using ISDC and LU for a representative malignant ovarian lesion. The leftmost panel displays the co-registered US-PAT image, with the white dashed quadrangle indicating the ROI. The middle column presents the ${\%\mathrm{sO}}_{2}$ map obtained using ISDC, and the right column shows the difference map $\Delta {\%\mathrm{sO}}_{2}$ (ISDC–LU), highlighting the improvement in oxygenation estimation. Below, THb maps derived from LU and ISDC illustrate spatial distributions of total hemoglobin concentration. The ISDC method compensates for depth-dependent fluence attenuation, resulting in increased THb estimates in deeper regions. The bottom right plot shows the depth-wise mean THb profile, demonstrating that ISDC better preserves THb values at greater depths through effective fluence correction.

${\%\mathrm{sO}}_{2}$ and THb maps of a representative malignant lesion, comparing LU and ISDC, are shown in Fig. 8. Consistent with phantom studies, ISDC increases ${\%\mathrm{sO}}_{2}$ estimation by ∼5%. In addition, ISDC enhances THb values in deeper tissues through depth-wise fluence compensation, improving both the visualization and quantification of hemoglobin concentration at greater depths.

**Fig. 9:**
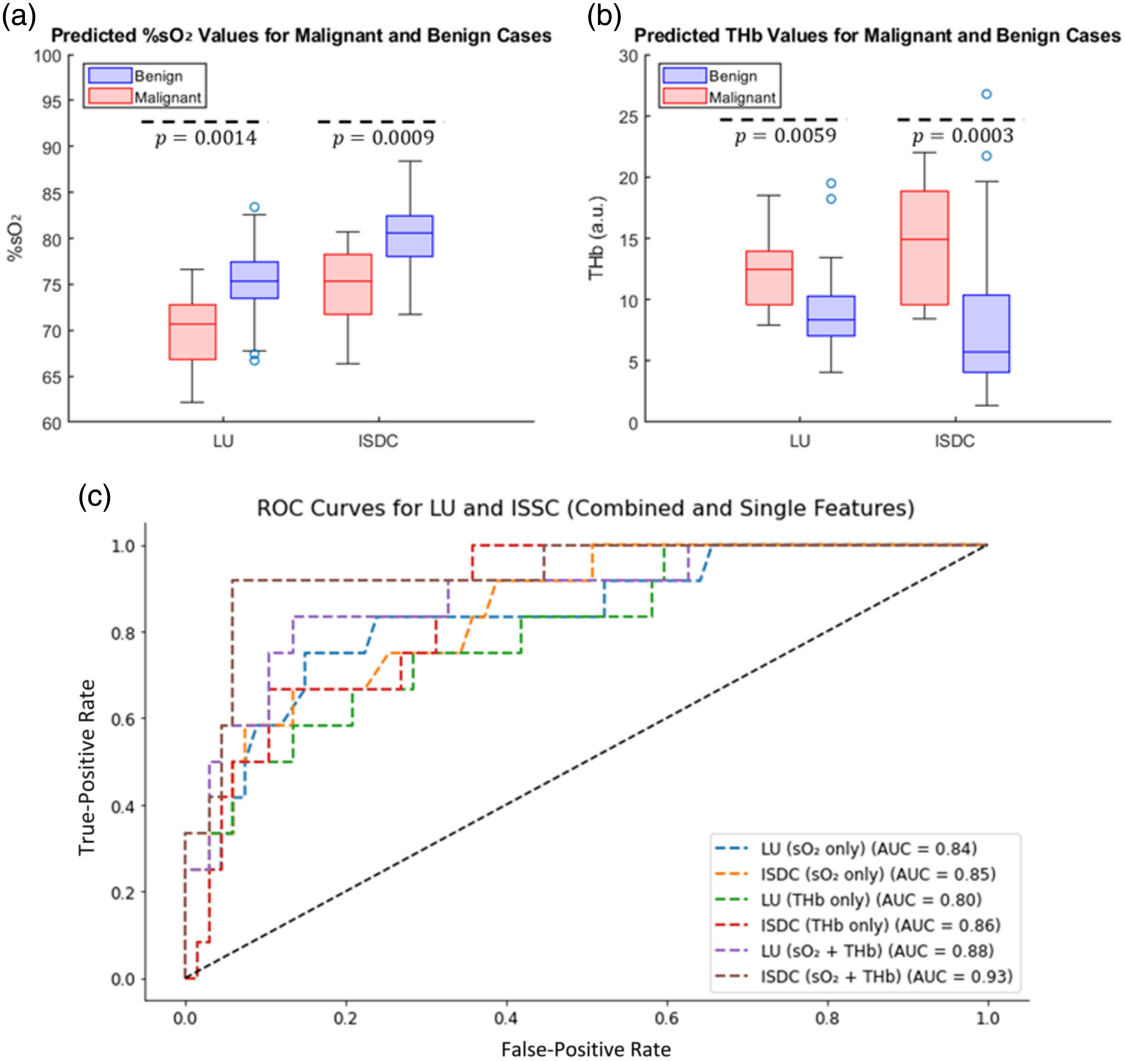
(a) Boxplots of mean ${\%\mathrm{sO}}_{2}$ values for 67 benign and 15 malignant ovarian lesions using LU and ISDC methods. The separation among groups improves with ISDC, as reflected by lower p-values ($1.4\times 10^{-3}$ with LU versus $9.3\times 10^{-4}$ with ISDC). (b) Boxplots of mean THb values for 67 benign and 15 malignant lesions. The THb ratio $R_{\mathrm{THb}}$ between malignant and benign cases increases from 1.4 (LU) to 1.9 (ISDC), indicating improved separation. The p-value between benign and malignant is reduced from $5.9\times 10^{-3}$ with LU to $3.0\times 10^{-4}$ with ISDC. (c) ROC curves for logistic regression models combining ${\%\mathrm{sO}}_{2}$ and THb or using individual features. The benign group includes normal and cyst lesions in this analysis. ISDC achieves the highest classification performance (AUC = 0.93) when combining both features, outperforming LU (AUC = 0.88) and single-feature models.

The statistical analysis of ${\%\mathrm{sO}}_{2}$ and THb values obtained with LU and ISDC for 15 malignant and 67 benign lesions is presented in Fig. 9. In Fig. 9(a), ISDC increases the estimated ${\%\mathrm{sO}}_{2}$ values by ∼5% in both groups, aligning them more closely with physiological expectations—typically 94% to 100% in arterial blood and ∼75% in venous blood.^24^ In Fig. 9(b), ISDC improves the THb separation between malignant and benign lesions. We define $R_{\mathrm{THb}}$ as the ratio of the mean THb values in malignant versus benign cases

$$R_{\mathrm{THb}}=\frac{{\overset{¯}{\mathrm{THb}}}_{\text{malignant}}}{{\overset{¯}{\mathrm{THb}}}_{\text{benign}}}.$$ (11)

$R_{\mathrm{THb}}$ increases from 1.4 with LU to 1.9 with ISDC, indicating enhanced contrast and improved diagnostic utility. This improvement likely arises from the combined effects of spectral compensation and depth-wise fluence correction, which together reduce wavelength- and depth-dependent biases, producing a more accurate THb distribution across the entire dataset.

To further assess classification performance, logistic regression models were developed using ${\%\mathrm{sO}}_{2}$ and THb as individual and combined features. As shown in Fig. 9(c), ISDC achieves the highest area under the receiver operating characteristic (ROC) curve (AUC=0.93) when combining both ${\%\mathrm{sO}}_{2}$ and THb, outperforming LU (AUC=0.88) and all single-feature models. For single features, ISDC also achieves higher AUCs than LU: 0.85 versus 0.84 for ${\%\mathrm{sO}}_{2}$, and 0.86 versus 0.80 for THb. The improved classification performance of ISDC is further supported by its consistently lower p-values, demonstrating stronger statistical separation between malignant and benign lesions.

These results demonstrate that ISDC not only enhances the physiological accuracy of ${\%\mathrm{sO}}_{2}$ and THb estimates but also significantly improves their value for lesion classification. Combined with its computational efficiency, ISDC is practical for integration into real-time US-PAT imaging systems for lesion diagnosis.

## 4 Discussion and Summary

We proposed and validated the ISDC approach that combines spectral fluence compensation and depth-wise fluence compensation to improve the ${\%\mathrm{sO}}_{2}$ and THb estimation in multispectral PAT. By addressing both wavelength-dependent and depth-dependent fluence variations, ISDC significantly improves the accuracy and reliability of quantitative PAT compared with conventional linear unmixing. In phantom experiments, CLU demonstrated comparable performance to the CC method, which serves as a reference standard, while outperforming LU in accuracy and stability. The ISDC approach further improved TSf estimation by correcting for depth-dependent fluence attenuation, resulting in more accurate measurements.

A practical trade-off also exists between the CC and CLU approaches. Although CC offers a more adaptive representation of wavelength-dependent fluence, it requires a larger number of wavelengths to stably estimate the combination coefficients. Under our four-wavelength clinical protocol, CC becomes vulnerable to system noise and measurement errors with inversion instability. CLU, using the pre-computed and numerically validated average spectrum $\phi_{\mathrm{ave}}$, offers greater robustness under spectral undersampling, noise, and measurement errors. Although not fully adaptive, CLU provides a stable and efficient correction that consistently improves over LU across the entire region of interest (ROI).

Applied to clinical ovarian data including 67 benign and 15 malignant lesions, ISDC increased ${\%\mathrm{sO}}_{2}$ values by ∼5% in both groups, aligning more closely with physiological expectations. Moreover, ISDC enhanced the separation between malignant and benign lesions in both ${\%\mathrm{sO}}_{2}$ and THb. For THb, ISDC enhanced contrast between malignant and benign lesions by increasing the ratio of mean THb values ($R_{\mathrm{THb}}$) from 1.4 (LU) to 1.9 (ISDC), reflecting stronger group distinction. To further assess diagnostic performance, logistic regression models were developed using ${\%\mathrm{sO}}_{2}$ and THb as input features. ROC analysis showed that ISDC outperformed LU across all configurations, achieving the highest AUC=0.93 when both features were combined (versus 0.88 for LU). When evaluated individually, ISDC also achieved higher AUCs for ${\%\mathrm{sO}}_{2}$ (0.85 versus 0.84) and THb (0.86 versus 0.80). Although the inter-lesion standard deviation of THb increased after ISDC, this likely reflects restoration of depth-related and patient-specific physiological variability. Future work will focus on systematically controlled experiments to support more refined calibration.

These findings underscore the potential of the ISDC approach to significantly improve the accuracy, robustness, and clinical utility of photoacoustic tomography in diagnosing and characterizing ovarian lesions. By providing more physiologically consistent ${\%\mathrm{sO}}_{2}$ and THb measurements and enabling clearer differentiation between malignant and benign cases, ISDC enhances the interpretability and diagnostic value of multispectral PAT.

The proposed ISDC has limitations. Although ISDC shows promise for improving ${\%\mathrm{sO}}_{2}$ and THb estimation, it relies on Monte Carlo simulations based on reported optical properties of various common tissue types, which may not fully capture individual patient variability. This assumption could limit the precision of fluence compensation in certain cases. In addition, the exponential depth-compensation model provides a simple and robust correction for depth-dependent fluence and acoustic attenuation; however, it remains a first-order approximation of a more complex physical process. Human ovarian tissue exhibits heterogeneous optical and acoustic properties, and the use of two global parameters $\mu_{f}$ and $\mu_{b}$ may not fully capture spatial variations in fluence and acoustic signal variation. As a result, residual depth-dependent bias may persist in certain regions, particularly in cases with atypical tissue composition or strong local heterogeneities. Furthermore, $\mu_{f}$ is estimated from digital phantoms based on reported population-averaged optical properties, which may not fully reflect patient-specific variability. These limitations may contribute to deviations in THb or ${\%\mathrm{sO}}_{2}$ quantification on a per-pixel basis, even though the global trends remain diagnostically useful.

Our current transvaginal US-PAT data acquisition imposes certain constraints, as the reduced spectral selection is used to minimize acquisition time and patient motion. This reduction may limit the richness of spectral information available for unmixing. In addition, the system remains susceptible to motion artifacts due to probe or patient movement during acquisition, potentially affecting image quality and quantitative accuracy. Future work will aim to optimize the US-PAT system data acquisition speed using an optical parametric oscillator laser for rapid tuning of the optical wavelength and to prospectively validate the diagnostic utility of ISDC in a larger patient cohort.

## Supplementary Material

10.1117/1.JBO.31.2.026002.s01

## Data Availability

The phantom data are available on GitHub: https://github.com/OpticalUltrasoundImaging/PAT_fluence_compensation.git. Patient data presented in this paper are not available but may be obtained from the corresponding author upon reasonable request.
