# Supplementary material for: Integrated spectral and depth compensation approach for optimizing oxygen saturation and total hemoglobin estimation in photoacoustic tomography for ovarian lesion diagnosis
Source: J Biomed Opt. 2026 Feb 4;31(2):026002. doi: 10.1117/1.JBO.31.2.026002 (PMC12869027; doi:10.1117/1.JBO.31.2.026002)
Supplement: Supplementary file 1 [file JBO_031_026002_SD001.pdf]

Supplementary Note S1 — Derivation of the Optimal Compensating Spectrum

In the Compensated Linear Unmixing (CLU) model, the compensating fluence spectrum $\phi_c(\lambda)$ is chosen to represent, with minimal bias, the ensemble of wavelength-dependent fluence spectra obtained from Monte Carlo simulations.

This section provides the mathematical derivation showing that the optimal compensator is the ensemble mean of the normalized eigenspectra.

Monte Carlo simulations on a digital phantom with physiologically relevant absorption and scattering coefficients were first performed to obtain wavelength-dependent fluence distributions $\phi(\boldsymbol{R}, \lambda)$. Each 3D fluence distribution was vectorized and subjected to dimension reduction to extract orthogonal fluence eigenspectra $\{\phi_1(\lambda),\ \phi_2(\lambda), \dots, \phi_n(\lambda)\ \}$, where n is the number of wavelength, which collectively describe the spectral variability due to different optical interactions including absorption and scattering. This eigenspectral set spans the range of fluence variations expected in the imaging configuration.

In the convex cone (CC) framework, the local fluence spectrum at a voxel is modeled as a non-negative combination of Monte Carlo-derived eigenspectra:

$$\phi_{\text{true}}(\lambda) = \sum_{i=1}^{n} \alpha_i \phi_i(\lambda), \qquad \alpha_i \geq 0 \tag{S1}$$

CLU aims to replace this adaptive combination with a single representative compensating spectrum $\phi_c(\lambda)$ that globally corrects wavelength-dependent bias in the linear unmixing process. The optimal compensator can be determined by minimizing the expected spectral error between ϕ_{true} and the candidate compensator:

$$\phi_c^\star = \arg \min_{\phi_c} \mathbb{E}\left[\min_{\alpha} \|\phi_{\text{true}} - \alpha\phi_c\|_2^2\right] \tag{S2}$$

where α accounts for wavelength-independent scaling due to factors including the Grüneisen parameter and illumination power.

Since linear unmixing (LU) depends on the shape rather than the magnitude of the fluence spectrum, both ϕ_{true} and ϕ_c can be normalized at the isosbestic reference wavelength ($800\ nm$):

$$\tilde{\phi}_{\text{true}} = \frac{\phi_{\text{true}}}{\phi_{\text{true}}(\lambda_0)}, \tilde{\phi}_c = \frac{\phi_c}{\phi_c(\lambda_0)} \tag{S3}$$

which fixes the scale to $\alpha = 1$ and simplifies Eq. (S2) to:

$$\tilde{\phi}_c^\star = \arg \min_{\tilde{\phi}_c} \mathbb{E}\left[\left\|\tilde{\phi}_{\text{true}} - \tilde{\phi}_c\right\|_2^2\right] \tag{S4}$$

Let $\mu = \mathbb{E}[\tilde{\phi}_{\text{true}}]$ be the ensemble mean of all normalized fluence spectra to be measured.

Expanding the expectation yields:

$$\begin{aligned} \mathbb{E}\left[\left\|\tilde{\phi}_{\text{true}} - \tilde{\phi}_c\right\|_2^2\right] &= \mathbb{E}\left[\left\|\tilde{\phi}_{\text{true}} - \mu + \mu - \tilde{\phi}_c\right\|_2^2\right] \\ &= \mathbb{E}\left[\left\|\tilde{\phi}_{\text{true}} - \mu\right\|_2^2\right] + \left\|\mu - \tilde{\phi}_c\right\|_2^2 + 2\mathbb{E}\left[\left(\tilde{\phi}_{\text{true}} - \mu\right)^\top\left(\mu - \tilde{\phi}_c\right)\right] \end{aligned} \tag{S5}$$

The cross term vanishes because $\mathbb{E}[\tilde{\phi}_{\text{true}} - \mu] = 0$, leading to:

$$\mathbb{E}\left[\left\|\tilde{\phi}_{\text{true}} - \tilde{\phi}_c\right\|_2^2\right] = \mathbb{E}\left[\left\|\tilde{\phi}_{\text{true}} - \mu\right\|_2^2\right] + \left\|\mu - \tilde{\phi}_c\right\|_2^2 \tag{S6}$$

The first term is independent of $\tilde{\phi}_c$, so the minimum is achieved when:

$$\tilde{\phi}_c^\star = \mu = \mathbb{E}[\tilde{\phi}_{\text{true}}] \tag{S7}$$

Thus, the optimal compensator under expected squared spectral-shape error is the ensemble mean of all normalized eigenspectra. In the absence of prior knowledge favoring any specific fluence spectrum within the set, we assume a uniform distribution:

$$\tilde{\phi}_c^\star(\lambda) = \frac{1}{n}\sum_{i=1}^{n} \tilde{\phi}_i(\lambda) = \tilde{\phi}_{\text{ave}}(\lambda) \tag{S8}$$

Because all eigenspectral in the paper are normalized, we can drop the tildes in implementation, yielding:

$$\phi_c^\star(\lambda) = \frac{1}{n}\sum_{i=1}^{n} \phi_i(\lambda) = \phi_{\text{ave}}(\lambda) \tag{S9}$$

Intuitively, if we express the true spectrum as $\phi_{\text{true}} = \phi_{\text{ave}} + \delta$ with zero-mean perturbation $\mathbb{E}[\delta] = 0$, then using $\phi_c = \phi_{\text{ave}}$ cancels the first-order ensemble-average bias term in LU.
